# Testosterone therapy masculinizes speech and gender presentation in transgender men

**DOI:** 10.1038/s41598-021-82134-2

**Published:** 2021-02-10

**Authors:** Carolyn R. Hodges-Simeon, Graham P. O. Grail, Graham Albert, Matti D. Groll, Cara E. Stepp, Justin M. Carré, Steven A. Arnocky

**Affiliations:** 1 Department of Anthropology, Boston University, 232 Bay Stated Rd., Room 102-B, Boston, MA 02215 USA; 2 Department of Forensic Sciences, George Washington University, Washington, D.C. USA; 3 Department of Speech, Language, and Hearing Sciences, Boston University, Boston, MA USA; 4 Department of Otolaryngology − Head and Neck Surgery, Boston University School of Medicine, Boston, MA USA; 5 Department of Biomedical Engineering, Boston University, Boston, MA USA; 6 Department of Psychology, Nipissing University, North Bay, ON Canada

**Keywords:** Reproductive biology, Health care, Endocrine system and metabolic diseases

## Abstract

Voice is one of the most noticeably dimorphic traits in humans and plays a central role in gender presentation. Transgender males seeking to align internal identity and external gender expression frequently undergo testosterone (T) therapy to masculinize their voices and other traits. We aimed to determine the importance of changes in vocal masculinity for transgender men and to determine the effectiveness of T therapy at masculinizing three speech parameters: fundamental frequency (i.e., pitch) mean and variation (*f*_o_ and *f*_o_-SD) and estimated vocal tract length (VTL) derived from formant frequencies. Thirty transgender men aged 20 to 40 rated their satisfaction with traits prior to and after T therapy and contributed speech samples and salivary T. Similar-aged cisgender men and women contributed speech samples for comparison. We show that transmen viewed voice change as critical to transition success compared to other masculine traits. However, T therapy may not be sufficient to fully masculinize speech: while *f*_o_ and *f*_o_-SD were largely indistinguishable from cismen, VTL was intermediate between cismen and ciswomen. *f*_o_ was correlated with salivary T, and VTL associated with T therapy duration. This argues for additional approaches, such as behavior therapy and/or longer duration of hormone therapy, to improve speech transition.

## Introduction

Transgender individuals describe the incongruence between their assigned sex at birth and their own gender identity to be a significant source of distress^1–3^. Compared to cisgender individuals, trans individuals have higher rates of suicide and suicide attempts^4^, distress^5,6^, depression, and anxiety^7^ and are more likely to be the victims of harassment and violence^8^. As a result, many seek gender confirmation surgeries or testosterone (T) therapy to bring their physical appearance and/or speech into alignment with their experienced gender. These interventions are generally effective: recipients report greater external validation of their gender from social engagements following treatment^9^ as well as overall improvements in quality of life^10–12^. For transgender men (referred to as transmen throughout) undergoing T therapy, more masculine speech is correlated with greater reported well-being^2^.

Two key acoustic characteristics of speech independently contribute to a masculine-sounding voice^13–16^: 1) fundamental frequency (*f*_o_) of vocal fold vibration, relating to voice pitch, and 2) the spectral structure of speech formants, which give identity to vowels and thus speech content, but also reflect vocal tract length (VTL)^17^. During puberty in cisgender males (whose natal sex and identity are both male; called “cismen” throughout), both the length and thickness of the vocal folds increase alongside T levels, thereby lowering *f*_o_^18–24^. The relationship between T and *f*_o_ remains consistent following puberty such that adult cismen with lower *f*_o_ also have higher salivary T^25–28^ (but not always^29^). Compared to ciswomen, cismen’s vocal folds are approximately 60% longer^30^ and speaking *f*_o_ is, on average, 80 Hz lower^31^. In addition to vocal fold changes, the larynx also descends in cismen during puberty, resulting in a larger and longer vocal tract. This 10–20% difference in VTL between cismen and ciswomen accounts for overall lower formant frequencies in cismen that occupy a smaller total frequency range^17,24,28,31^.

In previous studies of T therapy-related changes in the voices of transmen, *f*_o_ is reliably reduced in most participants after sufficient time on T therapy. Longitudinal studies demonstrate significant (e.g., ~ 60–70 Hz) and stable within-participant reductions in *f*_o_ after a minimum of ~ 3–4 months on T therapy^32–35^. Accordingly, participants typically perceived their own voices to be lower in pitch^32^ and participants with lower *f*_o_ reported a higher likelihood of being perceived as male over the phone^35^. Although T therapy often plays a significant role in female-to-male transition—noted by *f*_o_ values that are largely indistinguishable from those of cismen for many speakers^36^—not all individuals experience enough *f*_o_ lowering to reach the typical range of cismen. A meta-analysis found that 21% of patients fail to reach the *f*_o_ range of cismen after one year on T therapy^37^, by which time voice *f*_o_ lowering has typically reached an asymptote regardless of dose regimen^38^. Not surprisingly, an estimated 12–16% of patients are not fully satisfied with their vocal transition^32,37^.

Fundamental frequency (*f*_*o*_) is often reported to be the most heavily weighted cue for listeners in determining speaker gender identity^39,40^. However, studies of experimentally manipulated speech, in which *f*_o_ and formant frequencies are varied independently, reveal that listeners rely on more than just *f*_o_ when making judgments of speaker gender. For example, listeners can correctly identify ciswomen speakers as women even when their *f*_o_ values are artificially lowered to the range of cismen^41^. When *f*_o_ and formant frequencies are altered to the same perceptual degree (based on empirically derived just-noticeable-differences), listeners appear to rely more on formant information when making judgments about speaker body size, masculinity, and physical dominance^28,42^ (cf. Hillenbrand & Clark^15^). Thus, speech features other than *f*_o_ may impact the externally perceived gender identity of individuals undergoing the female-to-male transition, and these additional features, such as formant frequencies or the related measure of VTL, can potentially be used as outcome measures to assess efficacy of transition strategies and increase patient satisfaction. Yet, aside from one case study^43^ and an unpublished dissertation^44^, no studies on transgender men have investigated changes in formant measures with T therapy.

The present research has four central research questions. First, how important is masculinization of speech parameters relative to other traits for those undergoing a female-to-male transition? Second, is T therapy effective at masculinizing acoustic properties of speech that drive gender perception: *f*_o_ (mean and variation, *f*_o_-SD) and formant-based estimates of VTL? Research suggests that both are critical to perception of gender; however, little research exists on formant changes in transmen. Third, do higher salivary T levels and a longer duration of T therapy contribute to more masculine speech parameters? Finally, how do transmen rate their satisfaction with speech changes compared to other changes that occurred during T therapy?

## Results

### How important is masculinization of speech parameters for transmen undergoing T therapy?

**Figure 1:**
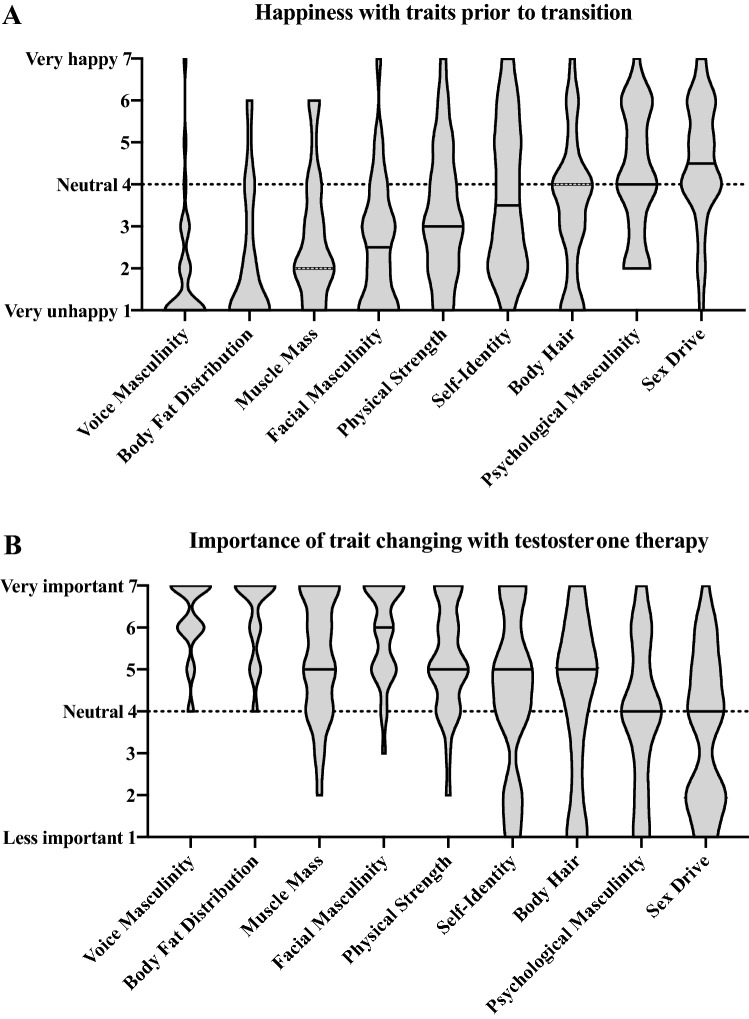
Satisfaction with traits prior to testosterone therapy (**A**) and importance of trait changing with testosterone therapy (**B**).

Voice masculinity was rated by participants as one of the traits they were least satisfied with prior to transition compared with all other traits (See Fig. 1A); 77% of participants rated voice masculinity as a “1” or a “2” on the 1-to-7 scale (1 indicated that they were extremely unhappy with the trait). Furthermore, when asked to rank the importance of observing change relative to other traits, change in voice masculinity was ranked as most important (see Fig. 1B); 83% of participants ranked it as a “6” or “7” (7 indicated that it was very important to see changes).

### Is T therapy effective at masculinizing acoustic properties of speech that drive gender perception?

**Figure 2:**
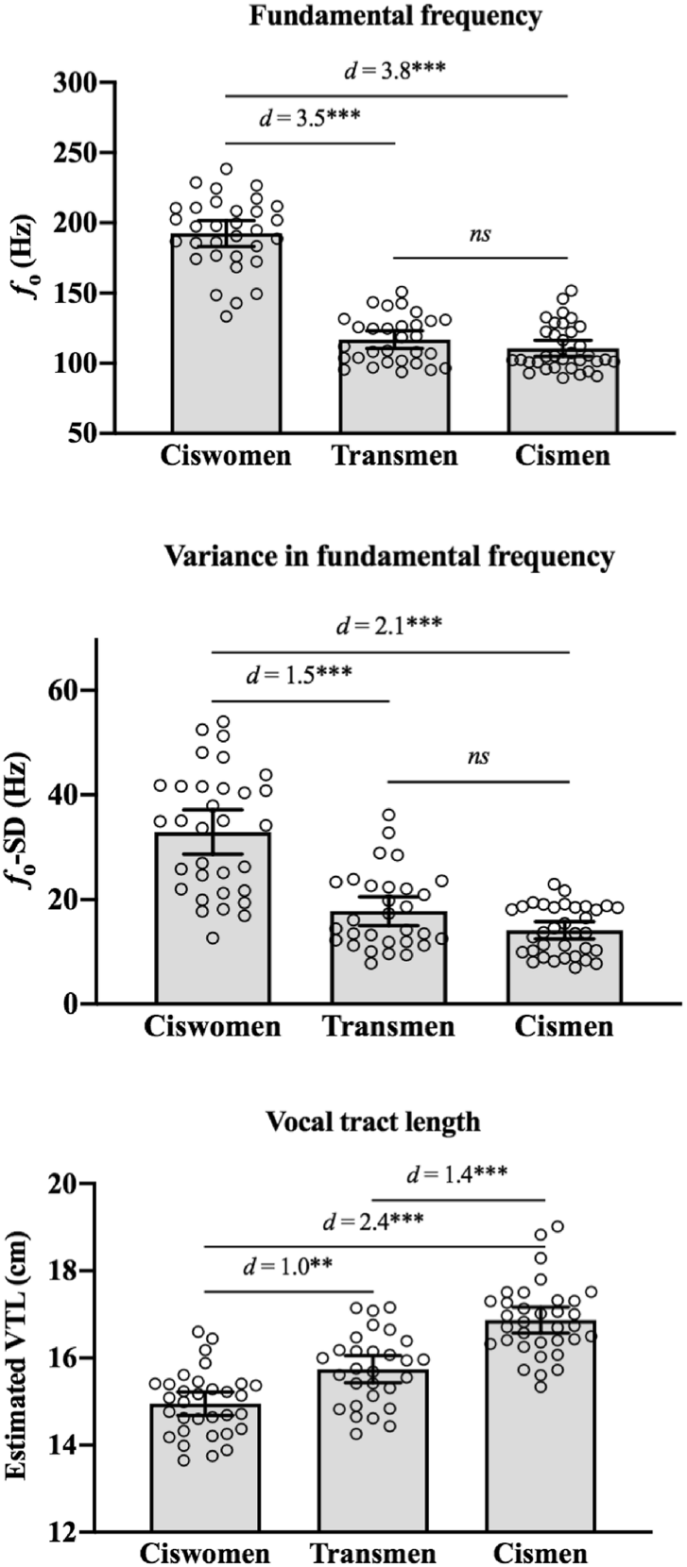
Fundamental frequency mean (*f*_o_) and variation (*f*_o_ –SD), and estimated vocal tract length (VTL) in ciswomen, transmen, and cismen speakers. *Note*: ** *p* < .01; *** *p* < .001. Fundamental frequency mean and variation were from the Rainbow Passage and vocal tract length was estimated from vowels /ɛ/ and /ɒ/. Error bars represent 95% confidence intervals.

Results suggest that T therapy is effective at masculinizing transmen’s *f*_o_ and *f*_o_-SD. A univariate ANOVA showed a significant effect of group on *f*_o_ [*F*(2, 93) = 164.8, *p* < 0.001, *η*^*2*^ = 0.78], *f*_o_ -SD [*F*(2, 93) = 45.4, *p* < 0.001, *η*^*2*^ = 0.49], and VTL [*F*(2, 92) = 47.5, *p* < 0.001, *η*^*2*^ = 0.51]. Post-hoc comparisons using Tukey’s HSD showed that the *f*_o_ and *f*_o_–SD of transmen were significantly lower than the *f*_o_ and *f*_o_–SD of ciswomen (Cohen’s *d* = 3.5 and 1.5, respectively), while indistinguishable from those of cismen. None of the transmen fell outside the cismen range for *f*_o_ (see Table 1); however, seven transmen (23%) had greater *f*_o_–SD than the highest value in the cismen group. Transmen’s estimated VTL was significantly longer than ciswomen (Cohen’s *d* = 1.0), but shorter than cismen (Cohen’s *d* = 1.4). Further, 23% of the VTL estimates were smaller in transmen than the lowest value in our cismen sample. See Table 1 for the mean, standard deviation, and range of speech parameters for transmen, cismen, and ciswomen groups. See Fig. 2 for visual comparisons of the three groups.

**Table 1:** Descriptive statistics for speech characteristics: Mean (± SD) and range.

	*f*_o_ (Hz)	*f*_o_ -SD (Hz)	VTL (cm)
Transmen (N = 30)	116.8 (± 16.9)	17.8 (± 7.4)	15.7 (± 0.8)
	93.7–150.9	7.8–36.3	14.3–17.2
Cismen (N = 34)	110.6 (± 16.6)	13.9 (± 4.8)	16.9 (± 0.9)
	89.5–151.8	6.9–23.0	15.3–19.0
Ciswomen (N = 32)	192.5 (± 25.7)	32.9 (± 11.8)	15.0 (± 0.7)
	133.4–238.6	12.6–54.1	13.7–16.6

*f*_o_ and *f*_o_ -SD measured from the Rainbow Passage. VTL estimated from vowels /ε/ and /ɒ/.

### Do higher salivary T levels and a longer duration of T therapy contribute to more masculine speech parameters?

**Figure 3:**
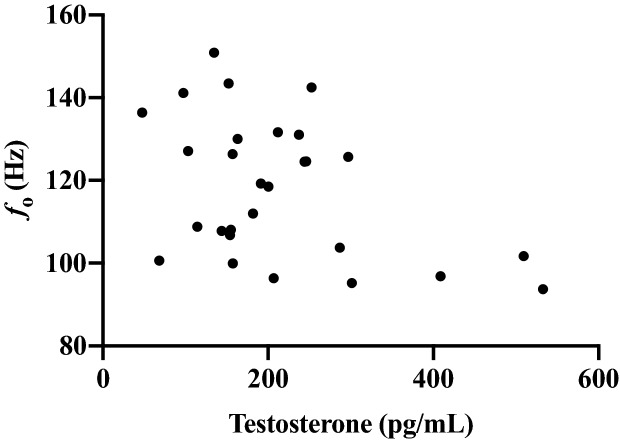
Association between salivary testosterone and fundamental frequency (*f*_o_).

**Figure 4:**
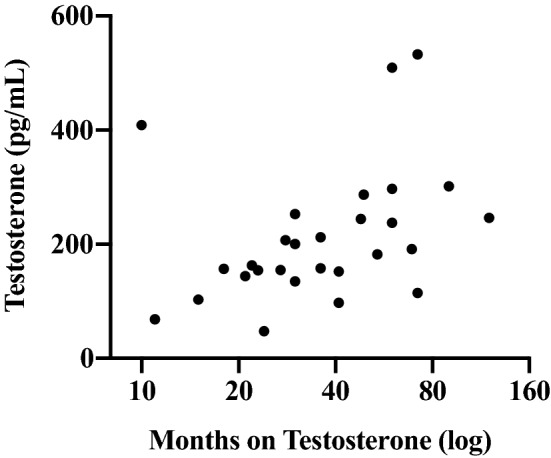
Salivary testosterone levels in transmen by time on T therapy.

Two individuals with very high T levels (2,889 and 794 pg/mL) were identified using Grubbs outlier test^45^ and excluded from the following analyses. Salivary T was significantly correlated with lower mean *f*_o_ (*r* = -0.37, *p* = 0.05), but not *f*_o_ -SD (*r* = -0.14, *ns*) or VTL (*r* = 0.03, *ns*). See Fig. 3. Testosterone levels were significantly correlated with time on T therapy; individuals who have been on therapy for a longer duration had higher T levels (*r* = 0.39, *p* < 0.05). See Fig. 4. Therefore, multiple regression models were then used to examine the independent contributions of circulating T and time on T therapy to each masculine speech parameter. Salivary T significantly predicted lower *f*_o_ (*ß* = -0.46, SE = 14.39, *t* = -2.23, *p* = 0.04), but T therapy duration did not (*ß* = 0.19, SE = 5.48, *t* = 0.90, *p* = 0.35). As a second step, we added the age at beginning of T therapy to the model. Only salivary T was associated with lower *f*_o_; however, it did not reach conventional significance levels (*ß* = -0.38, SE = 14.53, *t* = -1.83, *p* = 0.08). In a separate model predicting VTL, T therapy duration was a significant predictor (*ß* = 0.46, SE = 0.25, *t* = 2.30, *p* = 0.03), while salivary T (*ß* = − 0.09, SE = 0.67, *t* = -0.41, *p* = 0.69) and age at beginning T therapy were not (*ß* = 0.28, SE = 0.03, *t* = 1.49, *p* = 0.15). The model predicting *f*_o_-SD was not significant.

### How do transmen rate their satisfaction with self-perceived speech changes compared to other changes that occurred during T therapy?

**Figure 5:**
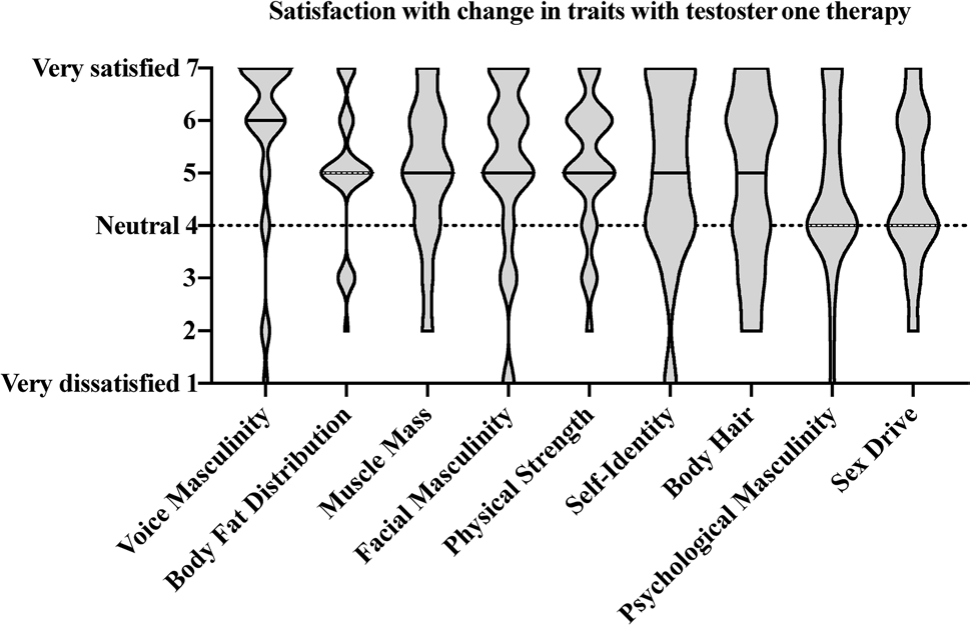
Satisfaction with perceived change in traits following testosterone therapy.

When asked to rate perceived amount of change following T therapy, voice masculinity was rated as the most changed among the surveyed traits with 72% of participants indicating that voice masculinity was “6” or a “7” on the 1-to-7 scale (7 indicated an enormous amount of change observed in the trait). When asked to rate satisfaction with perceived changes, voice masculinity was rated the highest among all survey traits with 77% indicating a “1” or a “2” (1 indicated that they were extremely satisfied with the changes). See Fig. 5.

## Discussion

Voice masculinization is particularly important to transgender individuals undergoing a female-to-male transition; compared with eight other masculinity traits, participants indicated that they were least satisfied with their voice prior to transition and ranked it highest in priority for seeing change. Further, after T therapy (which was effective at masculinizing *f*_o_ and *f*_o_ -SD in our participants), transmen were most satisfied with their vocal masculinization compared with other traits. Given its importance in a female-to-male transition and the growing number of individuals undertaking this treatment^46,47^, the need for evidence-based research on voice masculinization is high.

Our results show that, on average, T therapy is effective at masculinizing *f*_o_ and *f*_o_-SD. Transmen’s *f*_o_ values (mean and range) are comparable to those of cismen and statistically significantly lower than ciswomen’s *f*_o_ values. While we do not have recordings of these men prior to T therapy, we can assume that their *f*_o_ was close to the average for ciswomen and that their *f*_o_ has since changed by 3.5 standard deviations (or more^28^), which is nearly 80 Hz. Research suggests that 50% of listeners can detect shifts as low as 1.2 semitones (e.g., 7 Hz for a 100 Hz voice^42^); therefore, these changes likely have a strong impact on perception of gender. Overall, the current findings are consistent with previous results documenting substantial changes in *f*_o_ with T therapy in transmen^32–36^ and are suggestive of putative anatomical changes resulting from the action of T on the lengthening and thickening of vocal folds, similar to those occurring during puberty in natal males^19–24^. To understand the nature of these structural changes, future studies should use imaging techniques to objectively quantify vocal fold length and thickness at regular intervals during T therapy.

T therapy may not be sufficient for achieving formant frequencies that are indistinguishable from cismen. Our results showed that transmen’s estimated VTL was significantly longer than ciswomen but shorter than cismen. 23% of our participants’ VTL fell outside the range of our cismen sample, suggesting that T therapy alone does not fully masculinize larynx position. Despite research indicating that both *f*_o_ and VTL contribute to gendered voice perception^13–16^, only one other published study on transmen’s voice changes has examined VTL or formants^43^. This motivates development of additional treatments, such as behavioral therapy, to increase objective speech masculinity by increasing vocal tract length^48^. Previous studies on transmen’s speech changes have shown that most changes have occurred prior to 9 months of continuous T therapy^32–35,49^; however, these studies did not examine changes in estimated VTL. This study is the first, to our knowledge, to demonstrate statistical differences in VTL between samples of transmen and cisgender speakers [see Cler et al.^43^ for a single, detailed case study and Papp^44^ for an unpublished dissertation].

Incomplete masculinization of VTL (as well as *f*_o_–SD) may partly explain why 17% of our participants reported that they were ‘neutral’ to ‘extremely dissatisfied’ with changes in their vocal masculinity. This accords with previous studies showing 12–16% of patients are not fully satisfied with their vocal transition and 25% were still sometimes perceived as female on the phone^32,37^. Further, 31% expressed interest in further masculinizing their speech through additional treatments like behavioral voice therapy^32^. Despite the need for behavioral voice therapy among transmen, only one published study has examined its efficacy^48^. This is in contrast to transfeminine individuals where it has been shown to help individuals express their gender identity through speech, reduce gender dysphoria, and improve mental health and quality of life^50–53^.

Some of the acoustic properties of speech that drive gender perception were associated with features of T therapy. We found a significant inverse association between salivary T and *f*_o_; however, the results appear largely driven by 3 data points (see Fig. 3). Given the small sample size, it is unclear whether these individuals represent the normal range of variation. The association between current salivary T and *f*_o_ makes theoretical sense given the longer-term association between T administration and *f*_o_ change in transmen, the presence of androgen receptors on the vocal folds^54,55^, and the associations between T and *f*_o_ during puberty^18–23^. In addition, several studies have found links between salivary T and masculine vocal parameters in cismen^25,26,28^ (cf. Arnocky et al.^29^), and one study of cismen showed within-individual diurnal decreases in salivary T were associated with increases in *f*_o_^27^. Given the strong empirical and theoretical support for an association between T and *f*_o_, it is surprising that two previous studies on female-to-male speech changes^35,36^ did not find an association between serum T levels and *f*_o_.

Although there was not a significant association between salivary T and estimated VTL, T therapy duration was statistically significantly associated with VTL: longer T therapy durations were associated with longer estimated VTLs. This finding may suggest a longer-term relationship between T therapy and VTL. However, an alternative explanation is that this association reflects the confounding effect of time since transition given its close association with duration of T therapy. That is, even without formal voice training, transmen may be implicitly learning how to manipulate their vocal tract over time to achieve longer VTLs. Clinical studies on the relationships among dosing regimen, biological T availability, and speech parameters among transmen are necessary.

In summary, we see two important implications of these findings. First, a voice with a low pitch is a central aspect of masculine gender presentation because it is easily observable, highly sexually dimorphic, and difficult to approximate if not an adult male. Vocal fold dimorphism is one of the largest anatomical sex differences observed in humans (approximately 5 standard deviations^28,56^) and greater than any other extant ape^57^. Cisgender men and women differ by 60% in vocal fold length^30^ but only 8% in height^58^. Because vocal sexual dimorphism is extensive and, importantly, features little overlap in gender-typical vocal ranges, it is extremely difficult to speak in a voice consistent with the opposite sex, particularly in a sustained fashion^31^. These facts help explain why transmen are so dissatisfied with their *f*_o_ prior to T therapy. Similarly, our participants were also highly dissatisfied with body fat distribution, which is also very dimorphic^58,59^, easily observable, and difficult to change without hormonal therapy.

A second implication of these findings is that more research on speech changes in transgender males is necessary. The studies that have been published are limited by small sample sizes^32–34,49^, a lack of a control group for comparisons^32,33,35^, and a focus on only *f*_o_^34,49^. Additional research on T dosing regimens as well as the efficacy of behavioral voice therapy are particularly necessary. Better evidence-based treatments for transmen have health and safety repercussions. Transgender individuals are disproportionately targets of violence and being viewed as one’s gender is likely a critical component for safety^60–63^. Approximately 20–47% of transgender individuals have been physically or sexually assaulted and an additional 34–46% have been verbally threatened or harassed^62,64^.

In contrast to voice masculinization, participants did not place high importance on seeing an effect of T therapy on the non-physical trait “psychological masculinity”, highlighting participants’ dissociation between their own perception of gender and outward display of gender prior to therapy^65^. This incongruence is a source of extreme distress, which is associated with higher levels of depression, anxiety, substance abuse, and suicidal ideation and attempts among the transgender population—particularly those that have not begun to transition^5,7,9,65–68^. Receiving hormone treatment significantly improves mental health, social health, and physical health outcomes in transgender populations^2,7,9,66^. Vocal congruence contributes to these improvements; Watt et al.^2^ showed that more masculine voices significantly contributed to improved well-being and mental health in female-to-male transgender patients.

To summarize, this research was designed with several goals in mind. First, we aimed to quantify the importance of voice change—relative to other masculine traits—for transgender men undergoing testosterone therapy. No previous studies have explored this question, in spite of the strong interest in voice change among the transmasculine population. Our results show that voice masculinization is of central importance to transgender individuals undergoing the female-to-male transition compared with eight other masculine traits. Second, we asked whether T therapy was effective at masculinizing three gendered speech parameters. Our results show that, on average, T therapy is effective at masculinizing fundamental frequency mean and variation (*f*_o_ and *f*_o_-SD); however, transmen’s formant-based measure of vocal tract length (VTL) was significantly shorter than cismen. This study is the first, to our knowledge, to demonstrate statistical differences in VTL between samples of transmen and cisgender speakers. Third, we examined the association between salivary testosterone and vocal parameters. We found a significant inverse association between salivary T and fundamental frequency but no association with VTL. T therapy duration, however, was statistically significantly associated with VTL. These findings point to the need for more research on speech changes in transgender males—of particular importance are transition strategies that affect formant frequencies, which have largely been ignored in previous research.

## Method

### Participants

#### Transgender men

Participants included 30 individuals who were assigned female at birth; four of the participants identified their gender as non-binary and 26 identified as men. All were currently undergoing hormone replacement therapy with T for at least 9 months (*M*_months_ = 41.50, *SD* = 25.45) for the purpose of masculinization. Administration routes varied (intramuscular = 13, every 7–14 days; subcutaneous = 11, every 5–14 days; subdermal pellets = 4, every 3–4 months; transdermal = 1, daily). Ages ranged from 20–40 years old (mean: 25.9yrs ± 5.2) and the age when T therapy began ranged from 17 to 38 years old (mean: 22.4yrs ± 5.2); these were closely correlated with each other (*r* = 0.92, *p* < 0.001), but neither correlated significantly with time on T therapy or salivary T. Participants reported being primarily attracted to men (N = 6), women (N = 7), or both men and women (N = 15). Two participants declined to answer. Research suggests that lesbian women have lower *f*_o_ and *f*_o_-SD compared to heterosexual women (van Borsel et al., 2017); however, our sample size prohibited analysis by sexual orientation. Participants were excluded if they had been diagnosed with any vocal pathology or if they were habitual smokers in order to minimize the effect of potential spurious relationships. The ethnic composition of the sample was: Caucasian (86.7%), Asian (3.3%), Latin American (3.3%) and multiple ethnicities (3.3%). Recruitment was conducted via digital flyers and notifications on private or closed LGBTQ + or transgender Facebook groups, physical flyers posted at the 2017 Boston Pride festival, and word of mouth.

#### Cisgender men

Voice samples were collected from 122 cisgender male individuals. From this sample, we selected all individuals aged 21 and older to bring the mean age closer to that of the trans sample. The final N for cisgender men was 34. Ages ranged from 21 to 28 years old (mean: 23.0 years ± 2.2). More detail on this sample can be found in Arnocky et al.^29^.

#### Cisgender women

Participants included 32 undergraduate females from a larger study on immune function and phenotypic characteristics. From the larger data set, we selected all individuals 21 and older. Ages ranged from 21 to 32 years old (mean: 22.6 years ± 2.8). The sample was comprised primarily of Caucasian (94%) women.

### Procedure

All procedures were approved by the Boston University Institutional Review Board or the Nipissing University Research Ethics Board and were performed in accordance with relevant guidelines and regulations. Informed consent was obtained from all participants.

#### Questionnaire

Transgender participants answered a survey covering the following topics (7-point Likert scales, 4 = *neutral*): satisfaction with nine physical and/or psychological characteristics (see below) prior to transition (1 = *I was very unhappy*; 7 = *I was very happy*), importance of seeing changes in those characteristics during transition (1 = *Changes were not important to me*; 7 = *Changes were very important to me*), amount of observed change in those characteristics since starting T therapy (1 = *No change at all*; 7 = *Enormous amount of change*), satisfaction with this change since starting T therapy (1 = *I am very unhappy*; 7 = *I am very happy*), and overall satisfaction with their medical transition (1 = *I am very unhappy*; 7 = *I am very happy*). The surveyed characteristics were voice masculinity, facial masculinity (excluding facial hair), body hair, amount of muscle mass, physical strength, psychological masculinity, self-identity, sex drive, and body fat distribution.

#### Voice Recording

All speakers were asked to recite, in their normal speaking voice, the first sentence of the Rainbow Passage, numbers 1 to 10, and vowel sounds in the order /ε/ /i/ /ɒ/ /o͡ʊ/ /u/. Cis- and transmen’s voice samples were collected in a quiet room using an Audio-Technica AT4041 Cardioid Condenser Microphone connected to a Focusrite Scarlett 2i2 audio interface.

For the ciswomen sample, participants were asked to recite the same vowel sounds in the identical order described above. Voice samples were recorded in a sound attenuated room using a Neewer NW-700 condenser microphone with a 48 V phantom power supply and a pop filter. Participant voices were recorded using Goldwave version 6.31 software in mono with a sampling rate of 44.1 kHz and 16-bit quantization. The voice recordings were saved as high quality uncompressed wav files.

For all samples, recordings were analyzed using Praat version 5.3^69^ for mean *f*_o_ and standard deviation in *f*_o_ across the utterance (*f*_o_-SD) using the ‘voice report’ function. Pitch floor was set to 75 Hz and pitch ceiling was set to 300 Hz for cismen and transmen, and 100 Hz and 500 Hz for ciswomen, which are the recommended parameters for men’s and women’s voices, respectively. Otherwise, default settings were used. We used the Rainbow Passage for analyses involving *f*_o_ and *f*_o_ -SD; however, between-group comparisons (i.e., transmen vs. cismen and transmen vs. ciswomen) were similar when either vowels or counting were used for analyses. We also computed *f*_*o*_-CV (*f*_o_-SD / mean *f*_o_) following Pisanski et al.^70^ Transmen were not significantly different from either ciswomen or cismen in *f*_*o*_-CV; therefore, we only report *f*_o_-SD below.

In order to estimate VTL, formants were calculated using the acoustic recordings of the vowel sounds /ε/ and /ɒ/. All other vowels were omitted from VTL estimates due to narrow constrictions of the vocal tract (/i/) or lip rounding (/o͡ʊ/ and /u/) that complicate the relationship between VTL and formant frequencies^71^. Praat was used to generate a wide-band spectrogram of the acoustic signal, which was then used to calculate the first four formants using the standard formant tracking software in Praat. For each vowel, these automated formants were visually inspected, and the settings of the tracking software were adjusted until the formants aligned with the spectral representation of the signal. Formant values were calculated over the central stable part of the vowel. Third (F3) and fourth (F4) formant values for each participant were calculated by averaging the values from both vowels. These formant values were then used to calculate VTL estimates via Eq. (1)^72^, which shows an inverse linear relationship between formants and VTL that is derived from modeling the vocal tract as a uniform tube that is closed at one end (i.e., the vocal folds) and open at the other (i.e., the mouth). In Eq. (1), *n* is the formant number, F_*n*_ is the formant frequency (in Hz), and *c* is the speed of sound. Finally, VTL estimates from F3 and F4 are averaged for a single VTL estimate for each participant. F3 and F4 are used, because higher formants tend to be more stable and a better estimate of VTL^73^.

1 $${\text{VTL}} = \frac{{\left( {2{\text{n}} - 1} \right) \times {\text{c}}}}{{4 \times {\text{F}}_{\text{n}} }}$$

#### Saliva collection and analysis

Participants collected saliva samples in a 2-mL cryovial immediately upon waking the morning after they visited the lab to provide questionnaires and voice samples. Mean sample provision start time was 8:44 AM ± 1 h 59 min. Samples were refrigerated immediately after collection and then brought to the research team the following day where they were inspected using the Blood Contamination in Saliva Scale^74^. They were then stored in a -80 °C freezer until they were shipped overnight on dry ice for analysis. Samples were assayed in duplicate for free T using commercially available enzyme-linked immunoassay kits (DRG International, NJ, USA); average intra- and inter-assay coefficients of variation were 6.84% and 8.97%, respectively. For further detail on collection and assay of saliva samples, see Arnocky et al.^29^. T means and variance were significantly higher in transmen (M = 321.3 pg/mL, SD = 509, MIN = 47.7, MAX = 2889); however, when two outliers were removed (T = 784 and 2889 pg/mL), the trans mean (M = 212.8 pg/mL, SD = 116.8, MIN = 47.7, MAX = 538.2) was closer to the expected physiological range, compared with published averages for cismen (M = 152.1 pg/mL, SD = 60.5, MIN = 28.7, MAX = 152.0; values from Arnocky et al.^29^).

#### Data analysis

The following variables were log-natural transformed to address skew and increase normality: salivary T, time on T therapy, and *f*_o_. A univariate analysis of variance (ANOVA) was used to compare groups (transmen, cismen, and ciswomen) for each of the speech parameters (*f*_o_, *f*_o_-SD, and VTL). Initially, age was added to the model as a covariate and was then removed because it did not affect the analysis outcome. Linear regressions were used to examine associations between salivary T and speech parameters in transmen.
